# Effectiveness of a fully digital mandibular advancement appliance compared with CPAP in the treatment of obstructive sleep apnea

**DOI:** 10.1590/2177-6709.31.4.e262663.oar

**Published:** 2026-10-12

**Authors:** Taciana MORUM, Daniela SAMPAIO, Julia Badia HECKSHER, Jorge FABER

**Affiliations:** 1 Universidade de Brasília, Health Sciences School, Department of Dentistry (Brasília/DF, Brazil).; 2 Centro Universitário de Brasília, Health Sciences School, Department of Medicine (Brasília/DF, Brazil).

**Keywords:** Obstructive sleep apnea, Mandibular advancement appliance, CPAP, Digital workflow, Apneia obstrutiva do sono, Aparelho de avanço mandibular, CPAP, Fluxo de trabalho digital

## Abstract

**Introduction:**

Clinicians often face the decision of recommending Continuous Positive Airway Pressure (CPAP) or a Mandibular Advancement Appliance (MAA) for Obstructive Sleep Apnea (OSA).

**Objective:**

The objective of this study was to evaluate the effectiveness of a fully digital workflow MAA and compare its outcomes with CPAP therapy.

**Methods:**

Twenty adults with OSA confirmed by Type I polysomnography (AHI < 50 events/hour), non-adherent to CPAP, were enrolled in a crossover clinical study. After MAA titration (50% and 75% of maximum protrusion), participants underwent home sleep testing under three conditions: no treatment, CPAP, and MAA. Outcomes included oxygen desaturation index (ODI), snoring score, oxyhemoglobin saturation (SpO₂), hypoxic burden, and mean heart rate. Treatment-induced changes (Δ = treatment − no treatment) were compared using the Wilcoxon signed-rank test (α = 5%).

**Results:**

Both therapies significantly reduced ODI and snoring, compared to baseline. Median ODI decreased from 13.55 events/hour (untreated) to 6.05 with MAA and 5.30 with CPAP. No statistically significant differences were found between MAA and CPAP for ΔODI (p = 0.401), snoring (p = 0.103), hypoxic burden (p = 0.165), or heart rate (p = 0.824). A significant difference in SpO₂ was observed (p = 0.003), favoring CPAP; however, the difference was small.

**Conclusions:**

The fully digital MAA was effective in reducing oxygen desaturation, snoring, and hypoxic burden, showing no clinically meaningful difference compared with CPAP, supporting its use as a viable therapeutic alternative for OSA.

## RESUMO

**Introdução:**

Clínicos frequentemente precisam decidir entre o equipamento de Pressão Positiva Contínua nas Vias Aéreas (CPAP) ou Aparelho de Avanço Mandibular (AAM) para o tratamento da Apneia Obstrutiva do Sono (AOS).

**Objetivo:**

O objetivo deste estudo foi avaliar a efetividade do AAM confeccionado digitalmente e comparar seus resultados com a terapia CPAP.

**Métodos:**

Vinte adultos com AOS confirmada por polissonografia tipo I (IAH < 50 eventos/hora), não aderentes ao CPAP, participaram de estudo clínico cruzado. Após titulação do AAM (50% e 75% da protrusão máxima), os participantes realizaram teste domiciliar do sono sob três condições: sem tratamento, CPAP e AAM. Os desfechos incluíram índice de dessaturação de oxigênio (IDO), escore de ronco, saturação de oxiemoglobina (SpO₂), carga hipóxica e frequência cardíaca média. As mudanças induzidas pelo tratamento (Δ = tratamento − sem tratamento) foram comparadas pelo teste de Wilcoxon (α = 5%).

**Resultados:**

Ambas as terapias reduziram significativamente IDO e ronco, em comparação ao nível basal. A mediana do IDO diminuiu de 13,55 eventos/hora (sem tratamento) para 6,05 com AAM e 5,30 com CPAP. Não foram encontradas diferenças estatisticamente significativas entre AAM e CPAP para ΔIDO (p = 0,401), ronco (p = 0,103), carga hipóxica (p = 0,165) ou frequência cardíaca (p = 0,824). Houve diferença significativa - porém, pequena - na SpO₂ (p = 0,003), favorecendo o CPAP.

**Conclusões:**

O AAM digital foi eficaz na redução de IDO, ronco e carga hipóxica, não apresentando diferença clinicamente significativa em comparação ao CPAP, o que apoia seu uso como uma alternativa terapêutica viável para AOS.

## INTRODUCTION

Clinicians frequently face the decision of prescribing continuous positive airway pressure (CPAP) or a mandibular advancement appliance (MAA) for the management of obstructive sleep apnea (OSA). Both therapies are well-established and effective in improving symptoms and quality of life.^1^ ^-^ ^4^ However, given that MAAs demonstrate higher adherence rates and offer additional advantages such as portability, ease of use, and silent operation, the question remains whether CPAP should still be regarded as the gold standard for OSA treatment, since long-term adherence remains a major clinical limitation.

Treatment preference studies show that 29% of patients favor CPAP, 58% prefer MAA, and 13% opt for neither. Common factors contributing to low CPAP adherence include mask discomfort, dry mouth, anxiety, nightmares, claustrophobia, and insomnia. Nevertheless, both therapies demonstrate comparable improvements in daytime sleepiness, functional outcomes, and perceived health status.^5^

MAAs act by maintaining the mandible in a protruded position during sleep, increasing upper airway patency, and reducing apneic and hypopneic events.^6^ However, prolonged use may induce dental and occlusal changes, such as reductions in overjet and overbite, depending on appliance design.^7^ ^,^ ^8^ Therefore, an optimized design is essential to balance efficacy and side-effect profile.

Advances in digital Dentistry have enabled the development of fully digital workflows for the fabrication of customized MAAs.^9^ ^,^ ^10^ CAD-CAM technologies allow precise, reproducible, and efficient production of intraoral devices, potentially enhancing comfort and adaptation.

OSA impairs quality of life, causing excessive daytime sleepiness and fatigue, and is associated with an increased risk of diabetes and cardiovascular disease.^11^ ^-^ ^13^ MAAs improve clinical outcomes and represent an effective alternative for patients intolerant to CPAP therapy.^2^ ^,^ ^14^ Continued research is essential to further elucidate OSA pathophysiology and optimize treatment strategies.

Given the clinical relevance of adherence and technological innovation, the present study aimed to evaluate the effectiveness of a fully digital workflow MAA and to compare its therapeutic outcomes with CPAP in patients with OSA.

## MATERIAL AND METHODS

### STUDY DESIGN AND ETHICAL ASPECTS

The evaluation of the 3D-printed MAA effectiveness on polysomnographic parameters followed a randomized crossover design, in which all participants with obstructive sleep apnea (OSA) underwent home sleep testing under three conditions: no device, CPAP, and MAA, in a randomized sequence.

All participants were CPAP users before enrollment, but discontinued CPAP use at study entry and were treated exclusively with MAA throughout the follow-up period. CPAP was used only during the nights of home sleep testing, according to the randomized sequence, and not as a continuous treatment arm.

This study was conducted in Brasília, Brazil. The protocol was approved by the Research Ethics Committee of the *Universidade de Brasília* (CAAE 71031023.8.0000.0030) and registered in the Brazilian Clinical Trials Registry (RBR-3m46ncg). All participants signed informed consent.

### SAMPLE CALCULATION AND PARTICIPANTS

Sample size was calculated based on the Apnea-Hypopnea Index (AHI) reported by Uniken Venema et al.,^15^ adopting an effect size of 0.7918, α = 5%, and power = 80%, resulting in a minimum sample of 16 participants. Considering a possible 20% attrition rate, 20 individuals were included. Although oxygen desaturation index (ODI) was the primary outcome in the present study, previous evidence has demonstrated that ODI obtained from home sleep testing correlates strongly with AHI and may be used as a comparable marker of OSA severity.^16^ ^,^ ^17^

### INCLUSION CRITERIA

- Age 40-70 years.
- OSA confirmed by type I polysomnography - AHI < 50 events/hour.
- Intolerance or non-adherence to CPAP.
- At least 10 teeth per dental arch.

### EXCLUSION CRITERIA

- Body mass index (BMI) > 35 kg/m².
- Edentulism or insufficient appliance retention.
- Severe periodontal disease.
- Acute temporomandibular disorders.
- Anatomical airway obstruction.

### DIGITAL WORKFLOW AND APPLIANCE FABRICATION

Intraoral scans (iTero Element 2, Align Technology, Israel) were obtained for maxillary and mandibular dental arches. Interocclusal records were captured at centric occlusion, 50%, and 75% of maximum protrusion (Fig. 1). These digital records were used for appliance design and printing. 

Mandibular titration began with recording the protrusive bite for appliance fabrication. The titration process involved the following steps:

1. Measurement of horizontal overjet in centric occlusion and of maximum protrusive overjet using a custom millimeter ruler (Fig. 2).
2. Calculation of 50% of the maximum protrusion by summing the values obtained in centric occlusion and at maximum protrusion and dividing the result by two.
3. Calculation of 75% of the maximum protrusion by summing the value of maximum protrusion and the value corresponding to 50% of maximum protrusion, and dividing the result by two.
4. Assessment of the midline relationship between the maxillary and mandibular arches, maintaining the relationship present in centric occlusion during recording of advancement.
5. Digital bite registration at 50% and 75% mandibular advancement using a custom-made registration gauge designed to hold the mandible in the desired position during scanning (Gauge). The modified gauge was aligned parallel to the occlusal plane (Fig. 3).

**Figure 1:**
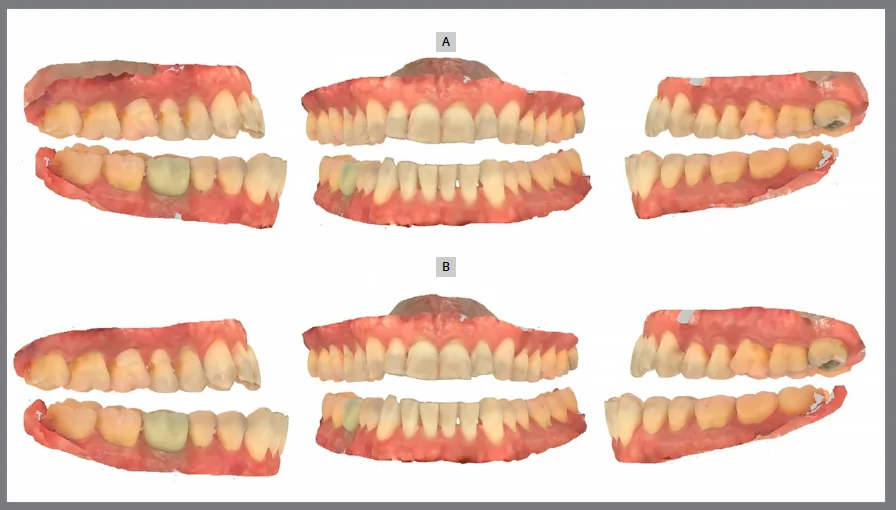
A) Mandibular advancement scan at 50% and B) 75% of maximum protrusion.

**Figure 2:**
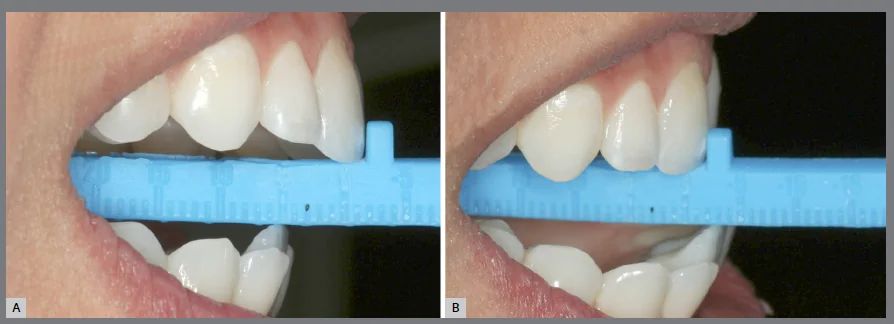
A) Measurement of horizontal overjet in centric occlusion and B) at maximum protrusion.

**Figure 3:**
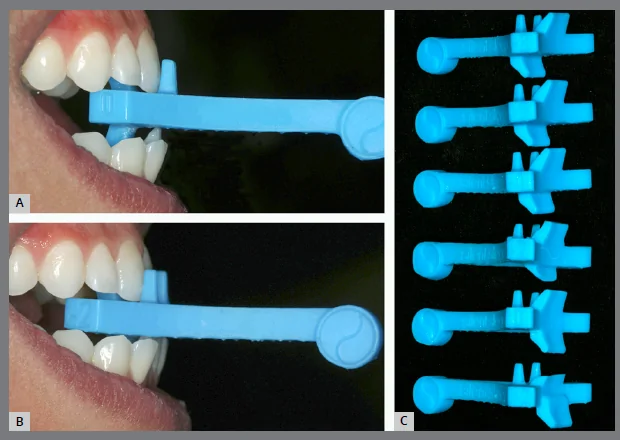
A) 50% maximum advancement registration using a 0 mm gauge. B) 75% maximum advancement registration using a -2 mm gauge. C) Digital gauges used for mandibular advancement recording.

The MAA consisted of two 3D-printed resin splints, one maxillary and one mandibular, connected by bilateral posterior connectors on the right and left sides to maintain the constructive bite position (Fig. 4). The splints covered the occlusal/incisal, buccal, and lingual surfaces of the teeth. Mandibular advancement was performed progressively by replacing the mandibular splint with another fabricated at a greater protrusive position.

**Figure 4:**
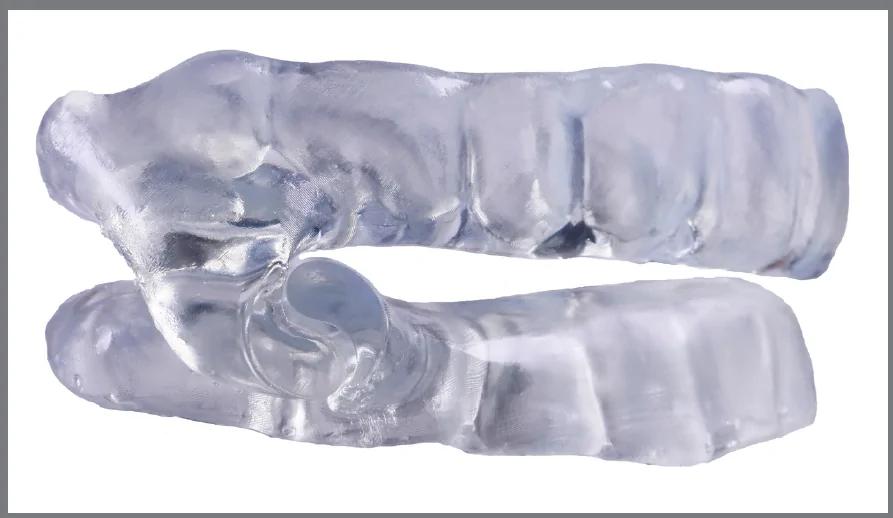
3D-printed mandibular advancement appliance.

A custom-made mandibular advancement gauge was developed to standardize bite registration during intraoral scanning, maintaining a 5-mm incisal distance and providing interocclusal space for appliance fabrication.

Once the customized intraoral device was printed with biocompatible resin, each participant was scheduled for the installation of the first mandibular advancement splint (Fig. 5). The appliance was adjusted to avoid excessive retention and to improve participant comfort. Participants received instructions regarding hygiene procedures, including daily cleaning with a medium-bristle toothbrush and liquid soap or mild detergent. Participants were instructed to use the appliance nightly for at least six hours during sleep. During the initial adaptation period, participants were allowed to remove the appliance during the night if discomfort occurred.

**Figure 5:**
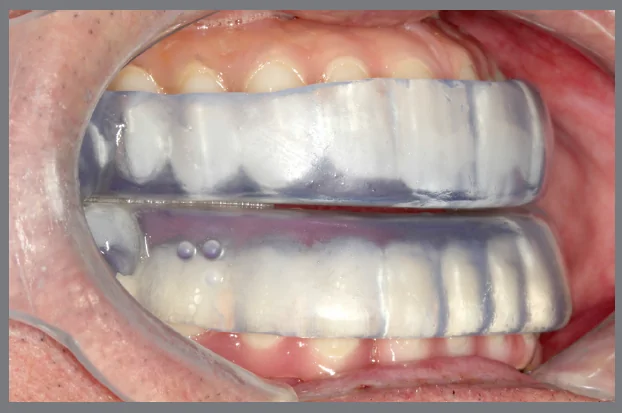
Installed mandibular advancement appliance.

Mandibular advancement was performed progressively by replacing the lower splint with another fabricated at a greater protrusive position. The interval between titration stages was approximately 30 days and could be extended in cases of temporomandibular discomfort or difficulty adapting to the appliance. After completion of the titration protocol, participants underwent home sleep testing while using the MAA during sleep.

### OUTCOME ASSESSMENT

Home sleep tests were performed using a portable monitoring device (Oxistar BX1, Biologix Sistemas, Brazil) under three conditions: no device, CPAP, and MAA. The examinations were conducted in the participants’ home environment during habitual sleep. Recorded parameters included:

1. Oxygen Desaturation Index (ODI) - the number of oxygen desaturation events per hour.
2. Snoring - the percentage of total sleep time spent snoring, and an arbitrary snoring score calculated based on intensity using the following formula: (silence × 0) + (low × 0.3) + (moderate × 0.7) + (high × 1.4).
3. Oxyhemoglobin Saturation (SpO₂) - the percentage of peripheral hemoglobin bound to oxygen in the bloodstream.
4. Hypoxic burden - the cumulative effect of oxygen desaturation events during sleep, quantified by the total duration and depth of hypoxemia (%minutes/hour below 90%).
5. Mean heart rate (HR) - beats per minute (bpm).

To account for potential order effects, participants were randomly assigned to different sequences of home sleep test assessments. Each person’s assignment was determined by simple randomization. This approach aimed to balance exposure to the interventions and reduce bias caused by the order of assessments. A one-week washout period was maintained between therapies.

For all analyzed variables, delta (Δ) values were calculated as the difference between the treatment and no-treatment conditions (Δ = treatment − no-treatment), separately for MAA and CPAP.

### STATISTICAL METHODS

Jamovi software (version 2.3.18) was used. Descriptive statistics were obtained from the analyzed variables. Normality was assessed by the Shapiro-Wilk test. The Wilcoxon signed-rank test was used to compare MAA and CPAP. Significance level was 5%.

## RESULTS

The flow of participants through the study is presented in Figure 6. Twenty participants completed all phases (14 men, 6 women). Mean age was 54.85 years; mean BMI 27.58 kg/m²; mean baseline AHI 28.88 events/hour.

**Figure 6:**
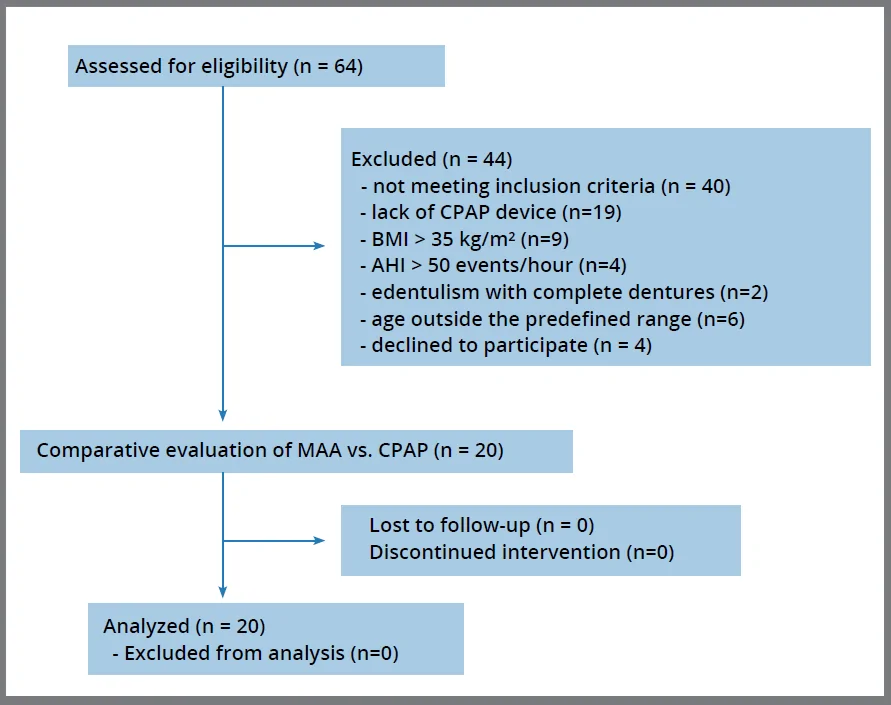
Flow diagram of study enrollment and analysis.

### ABSOLUTE SLEEP PARAMETERS

Descriptive statistics are shown in Table 1. In the untreated condition, the median ODI was 13.55 events/hour. Under treatment, median ODI decreased to 6.05/h with MAA and 5.30/h with CPAP, demonstrating clinically relevant improvement with both modalities.

**Table 1:** Descriptive statistics of the oxygen desaturation index (ODI - events/hour), snoring score (%), oxyhemoglobin saturation (SpO₂ - %), hypoxic burden (%min/hour), and heart rate (bpm) measured by HST (home sleep test) under three conditions: no device, MAA, and CPAP.

	No device	MAA	CPAP
	Median	Median	Median
ODI	13.55 (1.30- 63.00)	6.05 (0.40- 20.50)	5.30 (1.10- 24.60)
Snoring	28.20 (0.00- 116.00)	5.95 (0.00- 51.40)	0.70 (0.00- 53.90)
SpO_2_	93.50 (91.00- 95.00)	93.50 (91.00- 95.00)	95.00 (93.00- 96.00)
Hypoxic burden	59.55 (8.10- 192.40)	32.00 (0.90- 106.80)	23.15 (5.90- 111.00)
Heart rate	67.50 (48.00- 85.00)	66.00 (53.00- 92.00)	66.00 (50.00- 106.00)

Similarly, median snoring score decreased from 28.2% in the untreated condition to 5.95% with MAA and 0.70% with CPAP, indicating a substantial reduction in snoring with both therapies.

Mean SpO₂ stayed within normal physiological ranges across all conditions. Median SpO₂ was 93.5% in the untreated condition, remained at 93.5% with the MAA, and increased to 95.0% with CPAP.

Hypoxic burden also decreased from a median of 59.55%min/h in untreated conditions to 32.00%min/h with MAA and 23.15%min/h with CPAP, while mean heart rate was similar across all conditions.

### TREATMENT-INDUCED CHANGES (Δ ANALYSIS)

To allow direct comparison between therapies, treatment-induced changes were calculated (Δ = treatment − no treatment) and are presented in Table 2.

**Table 2:** Median, IQR and range of treatment-induced changes (Δ = treatment − no treatment) in sleep-related parameters under MAA and CPAP therapy.

	Δ MAA			Δ CPAP			p	95% CI
	Median	IQR	Range	Median	IQR	Range		
ODI (events/hour)	-6.25	13.2	-47.0; -0.4	-6.95	12.1	-46.3; 12.5	0.401	-0.80; 2.60
Snoring (%)	-17.5	37.8	-78.0; 25.0	-34.5	47.3	-84.0; 42.0	0.103	-1.00; 20.50
SpO_2_ (%)	0.0	0.5	-2.0; 2.0	1.0	2.0	0.0; 3.0	0.003	-2.00; -1.00
Hypoxic burden (%min/hour)	-27.0	35.4	-134; 14.4	-30.3	33.1	-161; 7.9	0.165	-3.95; 15.50
Heart rate (bpm)	0.0	6.0	-14.0; 9.0	0.0	6.0	-14.0; 23.0	0.824	-4.00; 2.00

Δ (delta) values were calculated as the difference between treatment and no-treatment conditions (Δ = treatment − no treatment) for all variables. Data is presented as median, interquartile range (IQR), and range. P-values were obtained using the Wilcoxon signed-rank test for paired comparisons between MAA and CPAP. Statistical significance was set at p < 0.05.

Both MAA and CPAP produced negative median Δ values for ODI, snoring, and hypoxic burden, indicating improvement compared to baseline. Median ΔODI was −6.25 for MAA and −6.95 for CPAP, with no statistically significant difference between therapies (p = 0.401). For snoring, median reductions were −17.5 (MAA) and −34.5 (CPAP), with no statistically significant intergroup difference (p = 0.103). Hypoxic burden decreased similarly with both treatments (p = 0.165), and no difference in heart rate was observed (p = 0.824).

A statistically significant difference was observed only for SpO₂ (p = 0.003), with CPAP demonstrating a slightly greater median increase. However, the magnitude of this difference was small and unlikely to represent a clinically meaningful advantage.

These findings indicate that the MAA achieved improvements comparable to CPAP in reducing respiratory events, snoring, and hypoxic burden.

## DISCUSSION

This crossover study demonstrated that a fully digital MAA significantly reduced oxygen desaturation index (ODI), snoring, and hypoxic burden, with no statistically significant differences compared to CPAP. 

The study was designed so that all participants were exposed to both CPAP and MAA, allowing within-subject comparisons. This approach was chosen to reduce interindividual variability, as each participant served as their own control. CPAP, previously used by all participants of the treatment group, was included as a comparator, while MAA was the primary intervention under investigation. This approach strengthens internal validity, reduces confounding, and increases statistical precision, compared with parallel-group designs.^18^

Carryover effects between interventions are unlikely to have significantly influenced the present findings, as both CPAP and MAA act only during active use and do not produce sustained remission of obstructive sleep apnea. A 1-week washout period was adopted to minimize any potential residual effects as had been suggested previously.^19^

OSA remains underdiagnosed despite its high prevalence, largely due to limited access to laboratory-based polysomnography.^20^ In this study, treatment efficacy was assessed using home sleep testing, a validated and cost-effective alternative for appropriately selected patients.^16^ ^,^ ^17^ Home-based diagnostics improve accessibility and patient acceptance by eliminating laboratory constraints and allowing assessment in a more natural sleep environment.

Although CPAP is considered the gold standard therapy because of its strong efficacy in reducing AHI, long-term adherence is frequently suboptimal. Discomfort, mask intolerance, nasal symptoms, and device-related inconvenience often lead patients to seek alternative therapies. The participants in this study had previous CPAP prescriptions but reported dissatisfaction, reflecting real-world clinical scenarios. Importantly, prior randomized trials have shown that despite CPAP producing greater reductions in AHI, overall health outcomes may be comparable to oral appliance therapy, due to higher adherence rates.^21^ ^,^ ^22^

In our study, the fully digital MAA reduced the ODI to a degree comparable to that achieved with CPAP, supporting digital fabrication as a clinically robust approach. Previous investigations have generally reported greater reductions in AHI with CPAP. For example, El-Solh et al.^5^ observed a significantly lower residual AHI with CPAP compared to MAA therapy. Similarly, in a meta-analysis by Zhang et al.,^11^ CPAP demonstrated superior efficacy in reducing respiratory events, although a higher proportion of patients expressed a preference for oral appliances. In contrast, Phillips et al.^21^, in a randomized crossover trial, reported smaller differences in AHI between therapies, with comparable improvements in daytime sleepiness and quality of life. These discrepancies likely reflect variations in appliance design, titration protocols, and patient characteristics. The effectiveness observed in the present study underscores the importance of individualized treatment planning and optimized appliance fabrication.

Advances in three-dimensional (3D) printing have accelerated the transition toward fully digital workflows in Dentistry. Digital fabrication enables high precision, reproducibility, and customization. The appliance used in this trial was metal-free and palateless, aiming to enhance comfort and facilitate oral hygiene. Discontinuation of oral appliance therapy is often associated with bulkiness, palatal coverage, metal components, allergic reactions, or fracture.^15^ ^,^ ^21^ ^,^ ^23^ Excessive palatal extension may compromise tolerance. Digital design flexibility may facilitate adherence, potentially through lighter and more ergonomic appliance designs. Although only one model was evaluated, the digital workflow itself represents a promising platform for future innovation in oral appliance therapy.

Recent evidence suggests that hypoxic burden is a stronger predictor of cardiovascular risk than AHI. Hypoxic burden quantifies the cumulative area under the oxygen desaturation curve, integrating depth and duration of events. Unlike AHI, which simply counts respiratory events, this metric reflects physiological severity. Azarbarzin et al.^24^ demonstrated that hypoxic burden was independently associated with cardiovascular mortality and incident heart failure, whereas AHI showed weaker associations. In the present study, both CPAP and MAA significantly reduced hypoxic burden, with no difference between treatments. This finding is clinically relevant, suggesting that oral appliance therapy may provide cardiovascular protection comparable to CPAP in CPAP-intolerant patients. Supporting this interpretation, randomized trials have shown that mandibular advancement therapy is not inferior to CPAP in reducing blood pressure.^21^ ^,^ ^25^

The degree of mandibular advancement plays a crucial role in therapeutic success. Lo Giudice et al.^10^ reported optimal effectiveness at approximately 75% of maximum protrusion, while 50% may be sufficient in mild OSA. Current guidelines from the American Academy of Sleep Medicine and the American Academy of Dental Sleep Medicine recommend gradual titration until symptom resolution or maximum tolerated advancement is achieved.^23^ Excessive protrusion may increase adverse effects such as temporomandibular discomfort, occlusal changes, or morning muscle fatigue. In this study, advancement rates of 50% and 75% were used to balance efficacy and tolerability.

Digital workflows further enhance customization of protrusion levels. Although the present protocol used predefined advancement percentages, digital design allows initiation at lower levels (e.g., 30%) in patients with joint discomfort and progressive titration up to higher levels if clinically required. Studies suggest that effective titration commonly ranges between 50% and 80% of maximum protrusion, underscoring the need for individualized adjustment.^12^ ^,^ ^22^ Evidence consistently supports the superiority of custom, titratable appliances over non-custom or fixed devices, particularly in moderate-to-severe OSA.^10^ Accordingly, a titratable digital device was selected in this study to permit personalized adjustment.

In clinical practice, lower CPAP adherence may attenuate its effectiveness in the real world. When adherence is considered, the therapeutic benefits of CPAP and MAA may converge. The absence of statistically significant differences in most outcomes in this crossover study suggests potential therapeutic equivalence in CPAP-intolerant patients. Fully digital MAAs may therefore represent a reliable and patient-centered alternative in OSA management.

Future studies with larger samples and longer follow-up are warranted to evaluate long-term occlusal stability, temporomandibular health, and cardiometabolic outcomes associated with digitally fabricated appliances.

## CONCLUSIONS

In this crossover sample of adults with OSA, the fully digital, titratable mandibular advancement appliance (MAA) produced clinically meaningful improvements and was statistically comparable to CPAP for treatment-induced changes in oxygen desaturation (ΔODI), snoring reduction, hypoxic burden, and mean heart rate. 

CPAP showed a statistically significant but small-magnitude improvement in oxyhemoglobin saturation compared with MAA. A fully digital MAA workflow-palateless, metal-free, and titratable via a structured 50%→75% advancement protocol-can be considered a pragmatic, effective therapeutic option for OSA patients who struggle with CPAP, supporting its adoption as an alternative pathway to reduce nocturnal desaturation and snoring, while improving overall hypoxic burden.

## Data Availability

Data restricted for ethical or legal reasons: Due to the sensitive nature of the data and ethical restrictions, the datasets supporting this study are not publicly available.
